# Design principles of chiral carbon nanodots help convey chirality from molecular to nanoscale level

**DOI:** 10.1038/s41467-018-05561-2

**Published:** 2018-08-24

**Authors:** Luka Ðorđević, Francesca Arcudi, Alessandro D’Urso, Michele Cacioppo, Norberto Micali, Thomas Bürgi, Roberto Purrello, Maurizio Prato

**Affiliations:** 1 Department of Chemical and Pharmaceutical Sciences, INSTM UdR Trieste, University of Trieste, Via Licio Giorgieri 1, 34127 Trieste, Italy; 2 Department of Chemical Sciences, University of Catania, Viale Andrea Doria 6, 95125 Catania, Italy; 3 CNR-IPCF Istituto per i Processi Chimico-Fisici, 98158 Messina, Italy; 4 Physical Chemistry Department, University of Geneva, 30 Quai Ernest-Ansermet, 1211 Genève 4, Switzerland; 5 Carbon Nanobiotechnology Laboratory, CIC biomaGUNE, Paseo de Miramón 182, 20014 Donostia-San Sebastián, Spain; 6 Basque Foundation for Science, Ikerbasque, 48013 Bilbao, Spain

**Keywords:** Nanoparticle synthesis, Nanoparticles, Synthesis and processing

## Abstract

The chirality of (nano)structures is paramount in many phenomena, including biological processes, self-assembly, enantioselective reactions, and light or electron spin polarization. In the quest for new chiral materials, metallo-organic hybrids have been attractive candidates for exploiting the aforementioned scientific fields. Here, we show that chiral carbon nanoparticles, called carbon nanodots, can be readily prepared using hydrothermal microwave-assisted synthesis and easily purified. These particles, with a mean particle size around 3 nm, are highly soluble in water and display mirror-image profile both in the UV–Vis and in the infrared regions, as detected by electronic and vibrational circular dichroism, respectively. Finally, the nanoparticles are used as templates for the formation of chiral supramolecular porphyrin assemblies, showing that it is possible to use and transfer the chiral information. This simple (and effective) methodology opens up exciting opportunities for developing a variety of chiral composite materials and applications.

## Abstract (web summary)

A promising and efficient route to chiral materials involves the transfer of chirality across length scales. Here, the authors use chiral molecular precursors to synthesize chiral carbon nanodots, which in turn can template the formation of chiral supramolecular assemblies.

## Introduction

Chirality is a symmetry description for any object, ranging from molecules to macroscopic items, that is not super-imposable on its mirror image. This geometric property is inherent in nature, as evidenced from homochiral proteins, nucleic acids and (poly)saccharides, which are also able to interact stereospecifically in a variety of biological events^1,2^. The appeal in understanding the physical, chemical and biological properties of chirality goes back to first observing optical activity in quartz (in 1811)^3^. However, the field of “art of chirality” started with Louis Pasteur that made the connection between chirality and optical activity with his remarkable experiments on tartaric acid crystals (in 1848)^4,5^. Subsequently, it was found that most biomolecules (proteins, nucleic acids, and saccharides) are chiral and, also to take advantage of their chiral recognition, admirable achievements have been made on the enantioselective synthesis of small molecules^6^. Almost two decades ago, the first inorganic chiral nanoparticles (NPs) were reported, employing stabilizing chiral ligands (glutathione) for gold nanoclusters^7^. While most studies are focused on metal nanoclusters (gold and silver), this approach has been extended to semiconductor nanocrystals, magnetic NPs and other nanostructures^8–16^. Chiroptical effects in metal and semiconductor NPs can be observed, besides the aforementioned interaction of the achiral nanoparticle with chiral molecules, by preparing inorganic nanostructures with chiral shape, achiral NPs that interact in a chiral configuration or NPs that possess intrinsically chiral cores^8–21^. Immense progress, in preparing and understanding nanoscale chiroptical materials, has been achieved and novel concepts and nanomaterials have opened up potential applications in catalysis, optics and biosensing, to name a few^8–12^.

The concept of introducing chirality in graphene quantum dots was recently applied by covalently attaching chiral moieties to their edges^22,23^. Although this elegant approach has produced graphene-based materials capable of interacting with cells^22^ or other molecules^23^ depending on the stereoisomer of the attached moieties, a novel methodology is needed to fully combine carbon nanomaterials with chiroptical properties. In this context, a one-step synthetic approach (that avoids cutting down of larger graphene sheets or carbon nanotubes and subsequent coupling with chiral molecules), capable of producing chiral carbon nanoforms with tunable optoelectronic properties, would be ideal. Herein, we report the synthesis of chiral carbon nanodots (CNDs), carbon nanoparticles characterized by sizes below 10 nm and fascinating fluorescence properties^24–26^. Additionally, they possess finer properties such as inexpensive and safe nature, and therefore could substitute the conventional semiconductor NPs, generally considered superior fluorescent materials^27–29^.

Herein we report a synthetic approach for preparing CNDs that exhibit chirality. The carbon dots are prepared through a multi-component microwave-assisted hydrothermal synthesis and we study their (chiro)optical properties. We also show that CNDs can form supramolecular complexes with chromophores (porphyrins) and that the chirality can be transferred, which highlights the emergence of novel possibilities.

## Results and discussion

### Design and synthesis

Carbon nanodots are generally considered carbon nanoparticles with a nanoscale carbon core covered by surface functional groups, such as carboxylic acids, alcohols, or amines^27–31^. Normally, these carbon nanoparticles can be prepared either by “top-down” cutting of larger carbon nanostructures or through “bottom-up” approaches, like one-step multi-component synthesis^32,33^. Especially the latter can make use of the different reactivity of the employed precursors, leading to the formation of defined and properly designed core and surface. For example, using bottom-up microwave-assisted hydrothermal synthesis, starting from abundant and non-expensive precursors such as arginine and ethylenediamine (EDA), it has been possible to readily prepare small and highly-fluorescent carbon nanodots^34^. Carbon-13 nuclear magnetic resonance, cyclic voltammetry, and chemical reactivity experiments unambiguously showed that arginine leads mostly to the formation of the core, while EDA is mainly responsible for the amino-rich surface^34–36^. However, during the hydrothermal microwave-assisted synthesis, as expected^37^, L-arginine undergoes racemization or consumption, resulting in achiral carbon nanodots (Supplementary Figure 1). We reasoned that, by appropriate choice of the surface precursor, i.e., a chiral diamine that retains chirality at high temperatures in water, chiral carbon dots could be prepared.

**Fig. 1:**
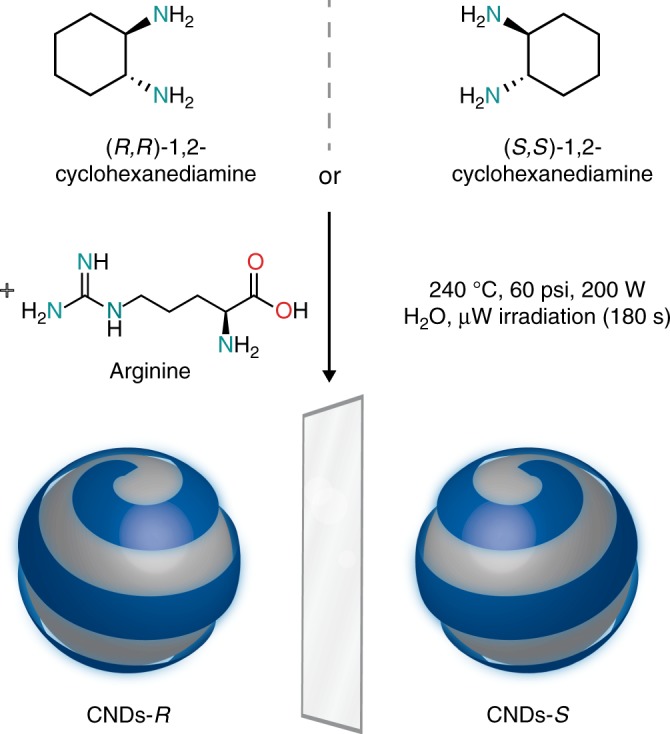
Design and synthesis of chiral carbon nanodots. Chiral CNDs were synthesized starting from (*R*,*R*)- or (*S*,*S*)-1,2-cyclohexanediamine and arginine, under hydrothermal microwave-assisted conditions (240 °C, 70–110 psi, 200 W for 180 s)

Chiral carbon dots were therefore prepared via microwave-assisted hydrothermal synthesis by using arginine, as the core precursor, and (*R,R*)- or (*S,S*)-1,2-cyclohexanediamine (CHDA)^38^, as the chiral surface precursors (Fig. 1). In the process of microwave heating (see Methods section for details), the solution changed color from transparent to dark brown as a result of the formation of nanodots. Large carbon nanoparticles were removed by filtration and the yellow filtrate was dialyzed against milli-Q water. The obtained nanodots, named CNDs-*R* or CNDs-*S*, are soluble in water (and other commonly employed polar organic solvents). For comparison, the achiral carbon dots prepared using arginine and ethylenediamine are named CNDs-EDA.

### Morphological and structural characterization

**Fig. 2:**
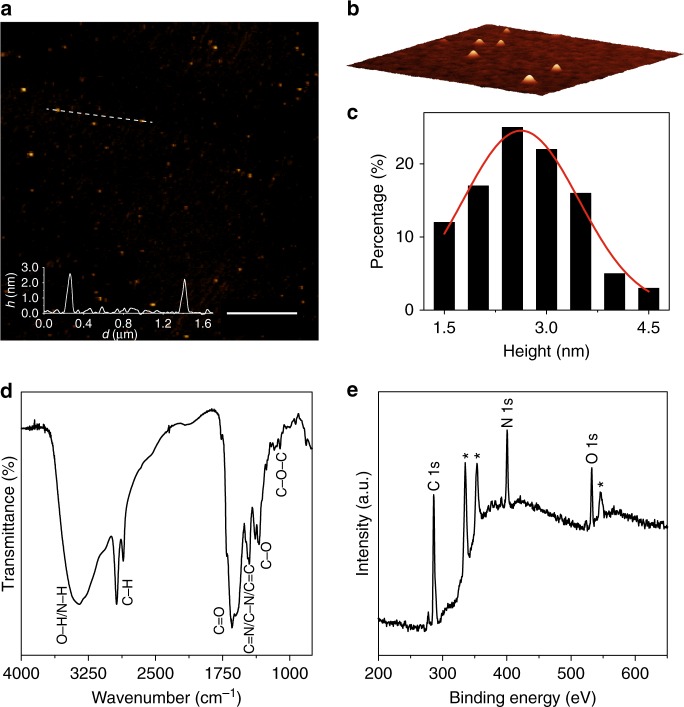
Morphological and structural characterization of CNDs-*R* and CNDs-*S*. **a** Tapping mode AFM (4.4 × 4.4 μm) from a drop-cast CNDs-*R* aqueous solution on a mica substrate (scale bar, 1 μm), inset is the height profile along the dashed line; **b** three-dimensional close-up AFM image (0.5 × 0.5 μm) of CNDs-*R*; **c** size histogram with curve fit using a Gaussian model; **d** FT-IR spectrum of CNDs-*S*; **e** XPS survey spectrum of CNDs-*S* showing the C1s, N1s, and O1s (deconvoluted spectra are in Supplementary Figure 4, *from Au substrate Au4d3, Au4d5, and Au4p)

The structure and the composition of CNDs-*R* and -*S* were determined by FT-IR spectroscopy and X-ray photoelectron spectroscopy (XPS), while morphological information were obtained by atomic force microscopy (AFM). As expected, the two chiral carbon nanodots show the same morphological and structural features (Fig. 2 and Supplementary Figures 2–4).

AFM images show the CNDs-*R* round shape and size of 2.64 ± 0.89 nm (FWHM: 2.09), comparable to the achiral CNDs-EDA^34^ (Fig. 2a–c and Supplementary Figure 2).

The CNDs-*S* FT-IR spectrum (Fig. 2d and Supplementary Figure 3) shows the presence of many functional groups, similar to the CNDs prepared using achiral EDA as amine precursor. Absorptions at 1174 and 1107 cm^−1^ can be attributed to C–O–C bonds, while absorptions at 1386, 1353, and 1342 cm^−1^ confirmed the presence of C–O bonds. Moreover, the absorption peaks at 1646, 1703, and 1766 cm^−1^ were indicative of C=O bonds, whereas the broad peak centered at 3350 cm^−1^ revealed O–H/N–H bonding. In addition, C–N/C=N/C=C (1491, 1470, and 1452 cm^−1^) functional groups can be identified, while peaks at 2935 and 2859 cm^−1^ can be related to the C–H bond stretching vibrations.

From the full-scan XPS spectrum of CNDs-*R* (Fig. 2e) C, N, O were detected (C1s at 286.2 eV, N1s at 400.6 eV and O1s at 532.3 eV). The atomic percentage for C, N, O are as follows: 65.9, 23.7, 10.4, respectively. To determine the C and N configurations in the CNDs-*S*, a detailed peak fitting analysis was performed, and the surface components are in good agreement with the observations from FT-IR experiments. The C1s spectrum of CNDs-*S* can be deconvoluted into five surface components corresponding to C=C at 284.8 eV (5.1%), C–C, and C–H at 285.8 eV (20.2%), C–O/C–N at 286.7 eV (56.8%), C=O/C=N at 288.5 eV (5.7%), as well as O–C=O at 289.6 eV (12.2%) (Supplementary Figure 4). The N1s spectrum of CNDs-*S* can be deconvoluted into four peaks centered at 398.7 (7.1%), 399.9 (25.7%), 400.9 (52.9%), and 401.9 (14.3%) eV corresponding to C=N, NH_2_, C–N–C, and N–C_3_ respectively. The presence of primary (and secondary) amino groups was confirmed by a positive Kaiser test, with an estimated value of 795 μmol g^−1^ of amino groups, which is in accordance to XPS measure.

Therefore, FT-IR and XPS measurements show that the surface of chiral nanodots contains similar multiple oxygen and nitrogen functional groups when compared to achiral CNDs-EDA, but with different contents.

### Photophysical and (chiro)optical properties

**Fig. 3:**
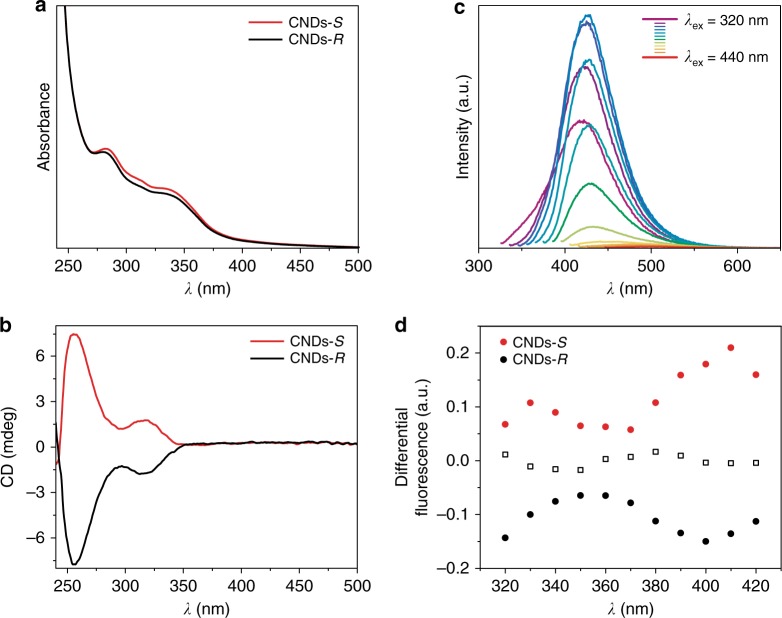
Photophysical and (chiro)optical characterization of CNDs-*S* and CNDs-*R*. Experiments performed in water at 298 K. **a** UV–Vis spectra of CNDs-*S* (red line) and CNDs-*R* (black line); **b** ECD spectra of CNDs-*S* (red line) and CNDs-*R* (black line); **c** FL emission spectra (at different excitation wavelengths) of CNDs-*R* (same emission profiles and intensities were observed for CNDs-*S*); **d** FDCD for CNDs-*S* (red circles), CNDs-*R* (black circles) and fluorescein (empty squares), measured using the experimental conditions reported in the Supplementary Methods, at the angle *θ* = 15°

The UV–Vis absorption spectra of CNDs-*S* and CNDs-*R* reveal two peaks at around 270 and 330 nm, which are related to the electron transitions from π (or n) to π* of C=C and C=O (Fig. 3a). The successful conferring of chirality from (*R,R*)- or (*S,S*)-CHDA to the carbon nanodots was confirmed by electronic circular dichroism (ECD) spectroscopy (Fig. 3b). The ECD spectrum of CNDs-*R* aqueous solution presents two negative Cotton effects at 260 and 320 nm, in accordance with the UV absorption bands (Fig. 3a). In the case of CNDs-*S*, inversion of the ECD signal was observed and indeed two positive Cotton effects at 260 and 320 nm were detected. In additional experiments we have prepared CNDs using various molar ratios of (*R,R*)- or (*S,S*)-CHDAs and the corresponding circular dichroism spectra showed that the optical activity of CNDs is dependent on the optical purity of the amine precursor (Supplementary Figure 5).

The fluorescence (FL) properties of the CNDs-*S* and CNDs-*R* show a broad emission peak at 425 nm when excited at the optimal excitation wavelength (350 nm) (Fig. 3c and Supplementary Figure 6). The fluorescence peak shifts from 419 to 466 nm when the excitation wavelength changes from 320 to 440 nm, with a slight excitation-dependent emission profile. These data reveal the different surface functional groups of CNDs-*S* and CNDs-*R*, compared to achiral CNDs-EDA. Indeed, the nanodot surface affects the FL properties since it determines the trapping of excitons under excitation. Furthermore, relative fluorescence quantum yields (FLQYs) were determined to be 20% and long average FL lifetimes were measured, around 8 ns for both CNDs-*S* and -*R* (double-exponential fit showed a short τ_1_ = 1.2 ns and a long τ_2_ = 11 ns component, Supplementary Figure 7 and Supplementary Table 1).

The chirality of the ground and excited states of CNDs was then probed through the detection of luminescence. The chirality of the ground state of CNDs was investigated through the fluorescence detection of circular dichroism (FDCD) by exciting the sample with circularly polarized light and measuring the fluorescence intensity. Opposite FDCD signals were measured for the two CNDs enantiomers. FDCD was measured for excitation wavelengths above 280 nm (Fig. 3d). Their opposite sign agrees with the opposite chirality observed in the electronic circular dichroism spectra. This result was not affected by fluorescence polarization effects or by polarized scattering; indeed, the differential fluorescence collected at the symmetric angle (*θ* = −15°) show the same spectral features (Supplementary Figure 8) and the contribution from the elastic scattering of CND was so low (i.e., the measured value of the excess scattered intensity in the visible wavelength range is almost comparable with pure solvent) that the longpass filter rejected it completely. Even placing an analyzer at the magic angle, despite the increased noise, did not change the spectral response. The negligible fluorescence polarization effects in the FDCD spectra can be led back to the long average fluorescence lifetime, which was about 8 ns for both CNDs (see Supplementary Figure 7 and Supplementary Table 1). During this time, the small size of CNDs allows for rotations fast enough to depolarize fluorescence emission (measured steady-state anisotropy = 0.03). Unfortunately, circularly polarized luminescence (CPL) spectra did not show any detectable chiroptical signal and thus no information about the excited state of the dots were obtained.

The wide emission spectral range of CNDs, extending above 600 nm, is constituted by different emission bands depending on the excitation wavelength (Fig. 3c). This occurrence indicates that CNDs possess many different dipoles absorbing light from UV to visible, which give rise to detectable fluorescence only if excited above 280 nm. As a consequence, chirality is observed in the deep UV region of the differential absorption spectrum with smaller and smaller amplitude at larger wavelength where the differential fluorescence signal becomes detectable.

**Fig. 4:**
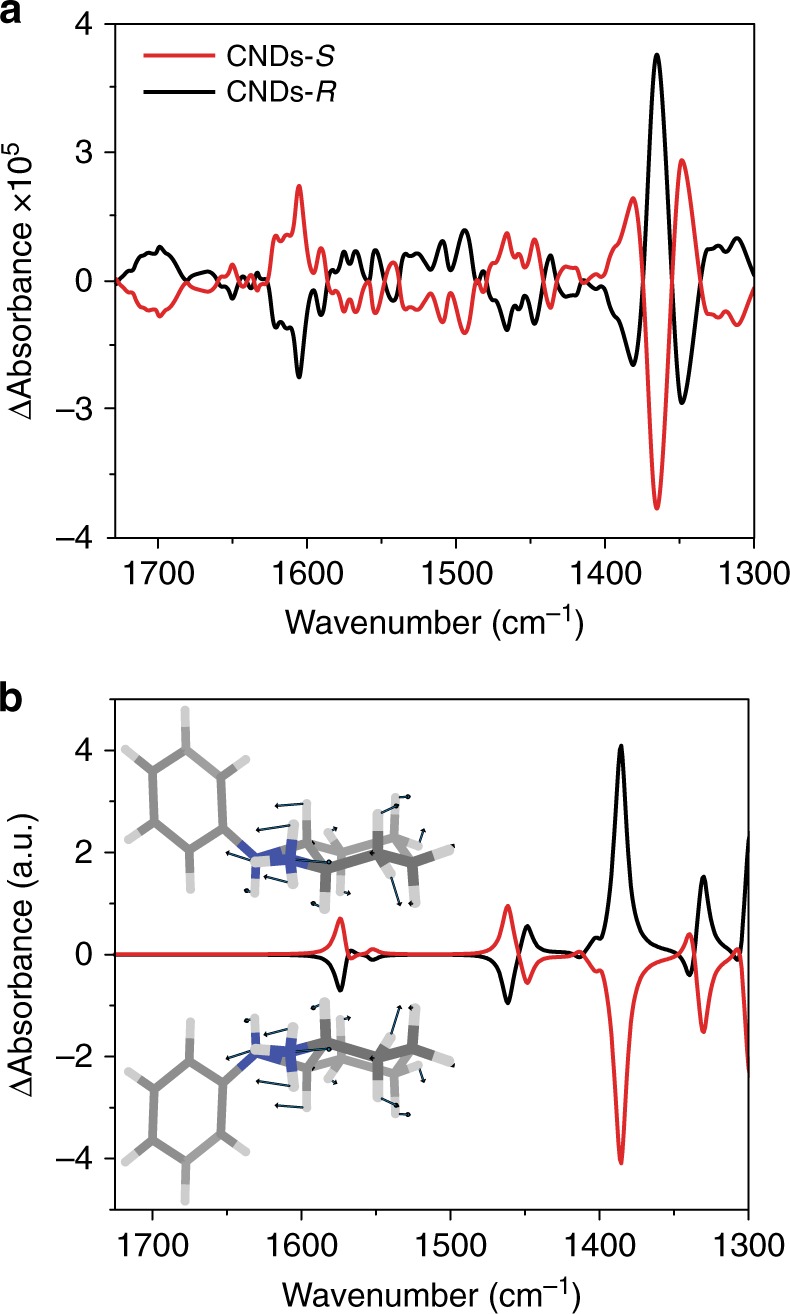
Comparison between experimental and calculated VCD spectra. **a** Experimental VCD spectra in water at 298 K of CNDs-*S* (red line) and CNDs-*R* (black line); **b** simulated VCD spectra obtained from DFT calculations of the cyclohexanediamine fragments (performed using the B3PW91 functional and a 6–31 G** basis set, water PCM solvation) with the insets showing the main displacement vectors for the computed chiral fragments

Finally, chirality in CNDs was investigated by vibration circular spectroscopy (VCD), a differential absorption of left and right circularly polarized IR light, which was previously employed in chirally capped metal NPs to obtain structural information^9^. The VCD spectra of aqueous CNDs-*S* and CNDs-*R* solutions show a mirror image relationship (Fig. 4a and Supplementary Figure 9), with stronger bands centered at around 1600 cm^−1^ and at slightly above 1350 cm^−1^. To extract structural information from the VCD spectra, the latter were simulated using different conformations of various cyclohexanediamine fragments (Supplementary Methods, density functional theory (DFT) calculations). DFT calculations on fragments, rather than entire molecules, were found appropriate when a suitable portion of the molecule was selected^39^ and consequently we focused on CHDA derivatives. As evidenced by simulated VCD spectra of the optimized structures (Fig. 4b), the stronger signals appear to be arising from primary amine N–H and cyclohexane C–H bending vibrational modes. Possibly the band around 1600 cm^−1^ has also contributions from C=O (amide) functional groups (Supplementary Figure 10). Though the system is simplified, these calculations are able to account for the strongest signals observed in the experimental VCD spectra and further support the notion of a chiral shell carbon nanoparticle, with the chirality originating from the CHDAs moieies incorporated on the carbon dots.

### Templating supramolecular assemblies

In order to investigate if it is possible to transfer the chiral information from the CNDs-*S* and CNDs-*R* to other molecules, we probed their non-covalent interactions with porphyrins, which are known to form donor-acceptor complexes with CNDs^40,41^. Porphyrins, square planar aromatic systems comprising 18 π electrons, are known for their structural robustness, absorption and emission properties and vast supramolecular chemistry.^42–44^ Moreover, porphyrin chemistry has advanced significantly so that the electronic and optical properties can be fine-tuned by shaping their periphery^45,46^. In addition, functionalization at the porphyrin periphery with charged substituents, gives access to water-soluble porphyrins, while maintaining the hydrophobic character of the macrocyclic core. This dichotomy can be exploited to drive the tendency of the porphyrins to self-assemble in aqueous solution and to modulate their aggregation onto other templates, inducing predefined architectures^47–49^. Furthermore, they are ECD silent, but binding to chiral molecules induces an ECD signal in their absorbance region, which can be regarded as a specific spectroscopic signature of the chirality of the “host” molecule^50^.

**Fig. 5:**
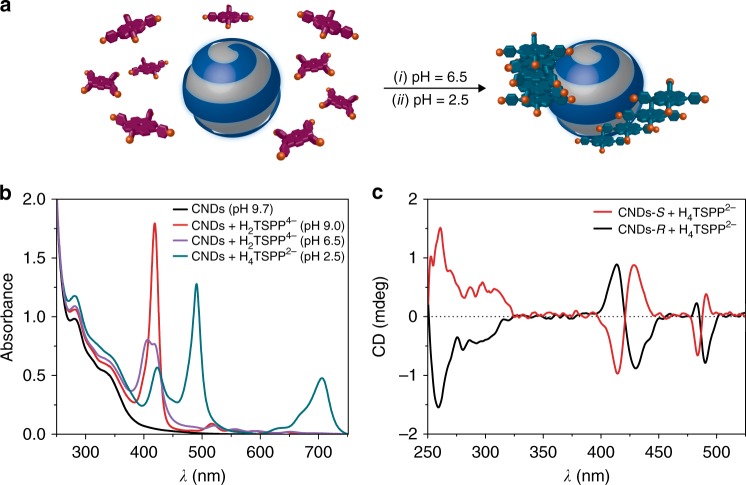
CNDs-*S* or CNDs-*R* as templates for chiral supramolecular aggregates. Experiments performed in water at 298 K. **a** Schematic representation of CNDs-*R* acting as chiral template after pH decrease trigger (purple = free base porphyrin, green = protonated porphyrin); **b** UV–Vis spectra of CNDs (black line) and CNDs + H_n_TSPP^m-^ solutions at different pHs (H_2_TSPP^4-^ concentration = 6.0 μM); **c** ECD spectra of CNDs-*S* (red line) and CNDs-*R* (black line), both in the presence of H_4_TSPP^2-^ (6.0 μM) at pH = 2.5

In order to establish electrostatic interactions with the amino groups around the CNDs-*S* or CNDs-*R* in aqueous solutions, we focused on the tetranionic *meso*-tetrakis(4-sulfonatophenyl)porphyrin (H_2_TSPP^4-^). Moreover, the partially protonated form (H_4_TSPP^2-^, p*K*_a_ = 4.8), being a zwitterionic molecule, is known to form both face-to-face (H-type, *λ*_max_ = 420 nm) and edge-to-edge (J-type, *λ*_max_ = 490 nm) aggregates^51–55^. These supramolecular aggregation processes are based on hierarchical self-assembly showing different thermodynamically and kinetically controlled paths, closely related to medium properties and experimental conditions (such as concentration, pH, and ionic strength)^56–58^. One of the most intriguing (and controversial) property of H_4_TPPS^2-^ aggregates is their unpredictable chirality. H_4_TSPP^2-^ aggregates should form a racemic mixture, exhibiting no optical activity^59^, however traces of chiral contaminants shift the 1:1 racemate equilibrium towards preferential chiral aggregates. To date only a few studies have been performed on the induction of preferential chirality to H_4_TSPP^2-^ aggregates using chiral molecules as templates^58,60^. Here we report the effect of chiral CNDs as inducer of chirality and controller of the size of H_4_TSPP^2-^ aggregates (Fig. 5a). A challenging aspect in these systems is the possibility of modulating the structure and properties of the final aggregates.

The addition of tetranionic H_2_TSPP^4-^ to a solution of CNDs-*S* or CNDs-*R* (at pH = 9.0) presents spectroscopic evidences of interaction, i.e., a hypochromic effect (H ~35%) and a slight broadening of the Soret band (Fig. 5b, red line). Since the surface of the CNDs is covered with amino groups, by changing the pH should be possible to tune the charge on the CNDs and therefore adjust the interactions with the porphyrin. Indeed, when decreasing the pH (from 9.0 to 6.5), we observ an intense hypochromic effect (H ~75%) and the splitting of the Soret band of the H_2_TPPS^4-^ (one more intense band at ~400 nm and the other at ~419 nm) (Fig. 5b, purple line). This experiment indicates the important role played by electrostatic interactions, indeed at pH 6.5 the porphyrin is still mainly tetranionic, whilst the positive charges on CNDs increase.

Then, in order to induce the formation of H_4_TSPP^2-^ aggregates, we further decreased the pH (to 2.5, using hydrochloric acid) of the solutions of H_2_TSPP^4-^•CNDs-*R* complex. The UV–Vis spectrum shows the complete formation of H-type and J-type H_4_TSPP^2-^ aggregates in less than 1 h (Fig. 5b, green line). The ECD band of the carbon nanodots decreases and two small induced bisignated ECD bands (centered at 420 and 490 nm) appear in the porphyrin aggregates absorption region (Fig. 5c, black line). Mirror image ECD spectra were acquired for the H_2_TSPP^4-^•CNDs-*S* complex (Fig. 5c, red line). The low intensity of the induced ECD signals of H_4_TSPP^2-^ aggregates might be due to a weak efficiency in transferring the chiral information from the CNDs to the porphyrin aggregates, owing to the decrease of the ECD bands of the carbon nanodots, at acidic pH. However, the size and the intensity of the communication of porphyrin aggregates also affects the ECD intensity and, indeed, resonance light scattering (RLS) measurments showed the formation of shorter porphyrin aggregates in the presence of CNDs (Supplementary Figure 11).

## Discussion

We report here on the design, synthesis and characterization of chiral carbon nanodots. We demonstrate that the chirality is conferred, through a hydrothermal bottom-up microwave assisted synthesis, by using arginine and *trans*-cyclohexanediamine. The obtained carbon nanodots possess similar size, morphological and photophysical properties to the previously reported CNDs. However, most importantly, they possess chirality that resulted in CNDs-*S* and CNDs-*R*. The chirality was investigated in their ground states and in the UV–Vis and IR regions. ECD spectroscopy show two mirror image profiles, also evidenced by their VCD spectra. Additionally, the origin of chirality is ascribed to the presence of numerous cyclohexanediamine moieties around the carbon-based amorphous core. Finally, the chirality was successfully transferred from the CNDs, through electrostatic interactions, to tetranionic porphyrins, inducing formation of chiral aggregates.

Although we believe that these findings will open up various possibilities and applications for carbon-based dots, there are also many possibilities to achieve this. In order to accomplish even further advances in catalysis, biology and nanotechnology, it might be necessary to prepare chiral assemblies of carbon dots or self-assembly of chiral supra-particles (as already achieved for semiconductor NPs^61,62^), decreasing the band gap^63^, exploiting quantum confinement effects^64,65^ or interacting differently with biological membranes^22^, to name a few. Such advancements could be fulfilled using properties reported here, and exploring new ones. Differently to other attempts at preparing chiral carbon nanoparticles^66–68^, the rationale brought forward in the present work, accompanied by proper purification and extensive investigation of the chirality, could be extended for preparing other types of carbon dots. By using our approach, chirality could be conferred from different chiral molecules^69^ to different core precursors^70^. For example, by using citric acid instead of arginine (and keeping CHDAs as chiral precursors) we have obtained, yet again, chiral carbon dots that display mirror-like images in the circular dichroism spectra, but possess different absorption and emission properties when compared to the arginine-based CNDs (Supplementary Figure 12). Therefore, future work should be directed at preparing and tailoring chiral carbon dots according to specific needs and applications.

## Methods

### Material synthesis

Chiral CNDs were obtained via microwave irradiation (CEM Discover-SP) of an aqueous solution of l-Arginine (Arg, Fluorochem, M03558) and (*R,R*)- or (*S,S*)-1,2-cyclohexanediamine (CHDA, Sigma Aldrich, 346721 and 346713). Typically, Arg (87.0 mg), (*R,R*)- or (*S,S*)-CHDA (57.0 mg) and milli-Q water (100.0 μL) were heated at 240 °C, 70–110 psi and 200 W for 180 s. In the process of microwave heating, the color changed from yellow to dark brown as a result of the formation of CNDs. The reaction mixture was then diluted with a few milliliters of water and the solution was filtered through a 0.1 μm microporous membrane separating a deep yellow solution that was dialyzed against pure water through a dialysis membrane for 2 days. The aqueous solution was then lyophilized giving a brownish solid (CNDs-*R*: 20.5 mg; CNDs-*S*: 22.0 mg).

### Chiral supramolecular assemblies

CND stock solution was prepared by dissolving 1.9 mg in 2.5 mL of ultrapure water (the pH of the solution was 9). The porphyrin selected for our study is the tetranionic *meso*-tetrakis(4-sulfonatophenyl)porphyrin (H_2_TSPP^4-^). The stock solution of porphyrin was prepared by dissolving a small amount of solid in ultrapure water at pH = 7, in order to obtain a concentration about 2 × 10^−4^ M, checked by spectrophotometer Jasco V-630 using *ε* = 4.8 × 10^–5^ M^–1^ cm^–1^ at *λ* = 413 nm. The sample solutions were obtained by mixing the CND and H_2_TSPP^4-^ solutions at pH = 6.5. After 30 min incubation, the pH was decreased to 2.5 in order to induce the formation of aggregates.

### Material characterization

Fourier-transform Infrared (FT-IR) spectra (KBr) were recorded on a Perkin Elmer 2000 spectrometer. Atomic force microscopy (AFM) images were obtained with a Nanoscope IIIa, VEECO Instruments. X-ray photoemission spectroscopy (XPS) spectra were measured on a SPECS Sage HR 100 spectrometer.

UV–Vis spectra were recorded on a PerkinElmer Lambda 35 UV–Vis spectrophotometer. Fluorescence spectra were recorded on a Varian Cary Eclipse fluorescence spectrophotometer. Time-resolved and differential fluorescence set-ups are detailed in the Supplementary Methods (and Supplementary Figure 13). Fluorescence time decay curve was fitted according to Supplementary Equation 1. ECD spectra were recorded using Jasco J-810. Resonance light scattering (RLS) measurements were acquired using a spectrophotometer FL11 Jobin Yvon Horiba. Vibrational circular dichroism (VCD) spectra were recorded on a Bruker PMA 50 accessory coupled to a Tensor 27 Fourier transform infrared spectrometer.

### DFT calculations

The geometry optimizations, vibrational frequencies, infrared absorption and VCD intensities were performed by DFT. The calculations were performed using the B3PW91 functional and a 6–31 G** basis set (water PCM solvation).

### Data availability

The authors declare that the main data supporting the findings of this study are available within the article and its Supplementary Information file. Additional data are available from the corresponding authors upon request.

## Electronic supplementary material

Supplementary Information
