# Supplementary material for: Design principles of chiral carbon nanodots help convey chirality from molecular to nanoscale level
Source: Nat Commun. 2018 Aug 24;9:3442. doi: 10.1038/s41467-018-05561-2 (PMC6109168; doi:10.1038/s41467-018-05561-2)
Supplement: Supplementary file 1 — Supplementary Information [file 41467_2018_5561_MOESM1_ESM.pdf]

Supplementary Information

Design principles of chiral carbon nanodots help convey chirality from molecular to nanoscale level

Luka Đorđević,* Francesca Arcudi,* Alessandro D'Urso, Michele Cacioppo, Norberto Micali, Thomas Bürgi, Roberto Purrello and Maurizio Prato*

Supplementary Figures

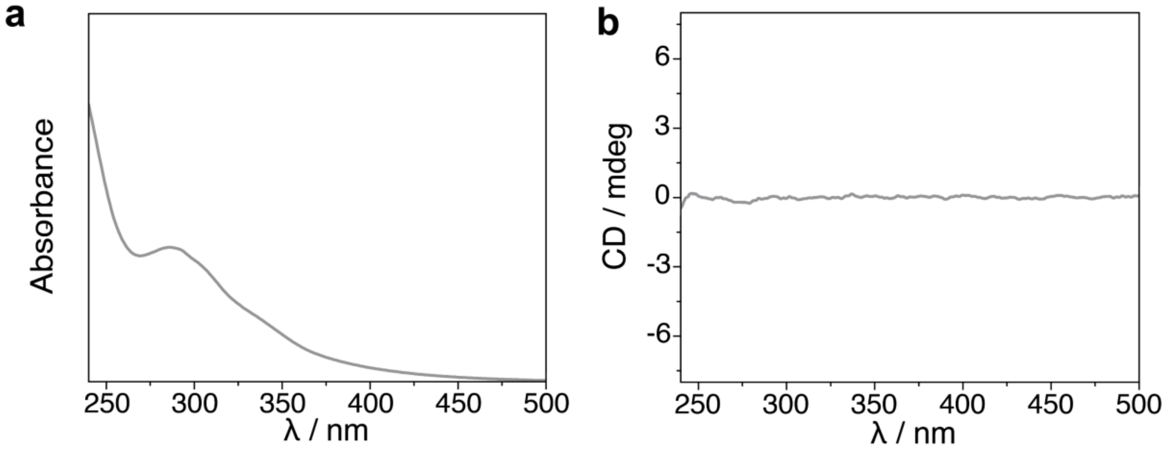

Supplementary Figure 1. **(Chiro)optical characterization of CNDs-EDA.** Experiments performed in water at 298 K. (**a**) UV-Vis spectra of CNDs-EDA (grey line); (**b**) ECD spectra of CNDs-EDA (grey line).

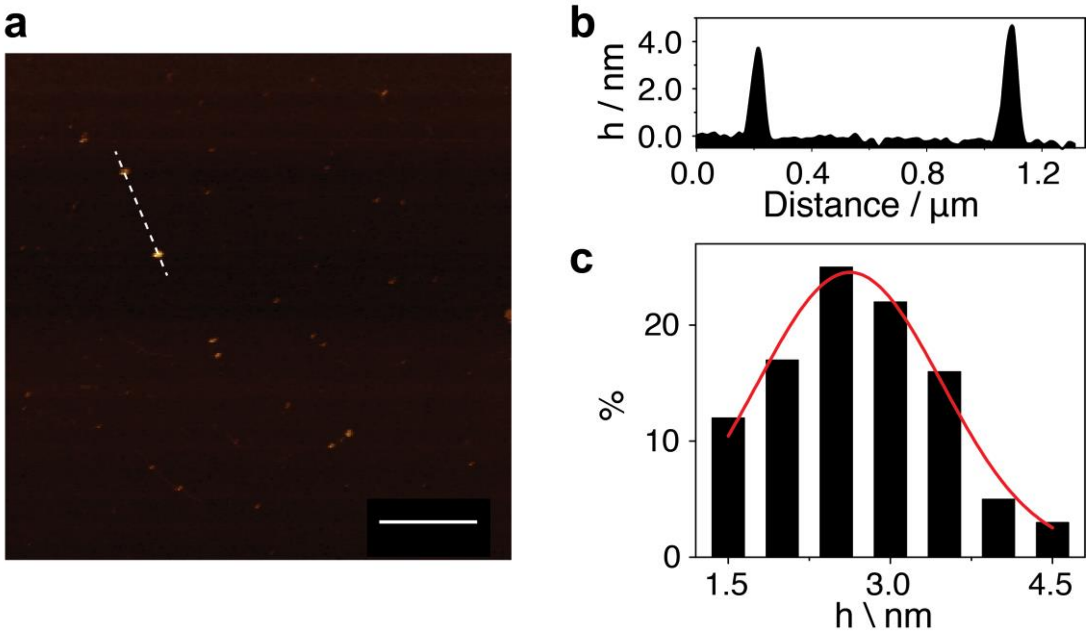

Supplementary Figure 2. **AFM of CNDs-*S***. (**a**) Tapping mode AFM (4.4 × 4.4 µm) from a drop-casted aqueous solution on a mica substrate (scale bar, 1 µm); (**b**) height profile along the dashed line; (**c**) size histogram with curve fit using a Gaussian model.

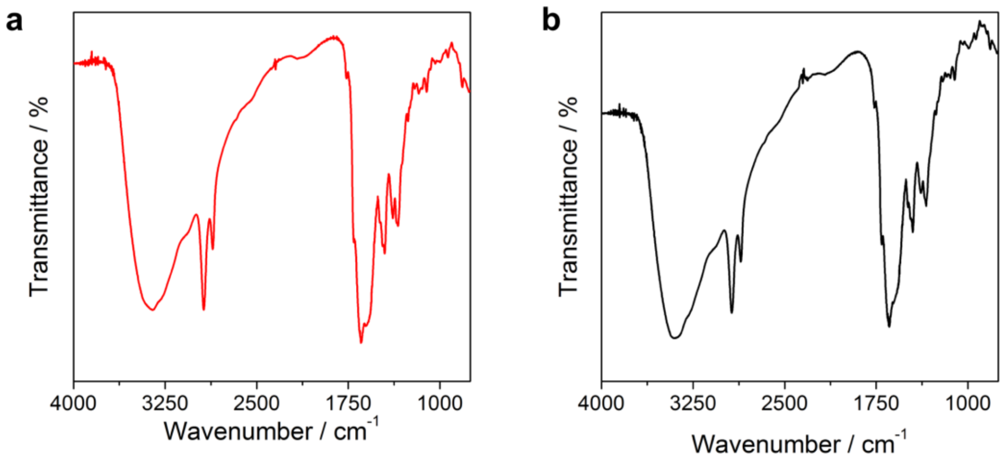

Supplementary Figure 3. **FT-IR spectra**. (**a**) CNDs-*S* and (**b**) CNDs-*R*.

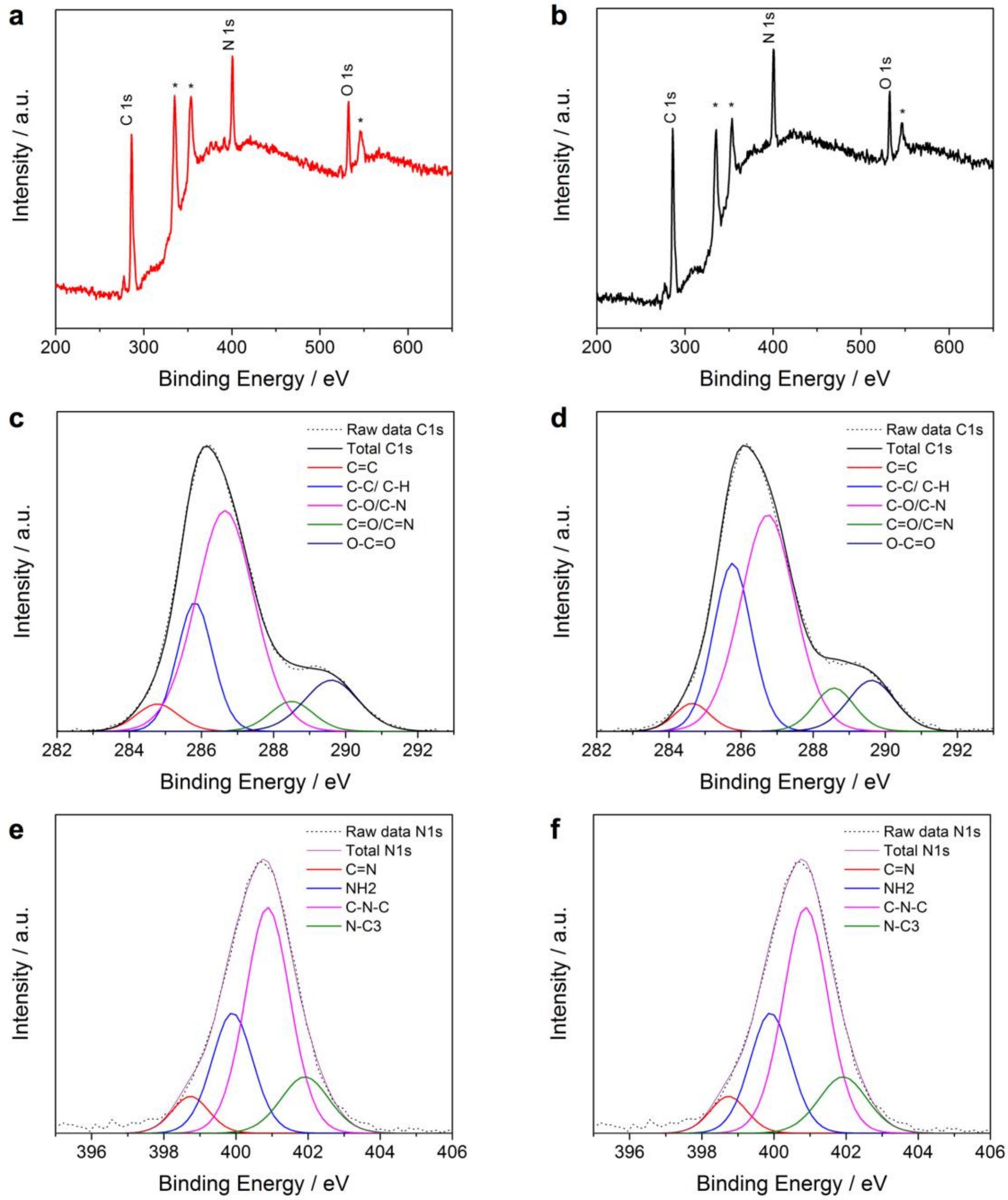

Supplementary Figure 4. **XPS of CNDs.** CNDs-*S* (left) and CNDs-*R* (right). (**a,b**) XPS survey showing the C1s, N1s and O1s (*from substrate Au4d3, Au4d5 and Au4p); (**c,d**) deconvoluted C1s spectra and (**e,f**) deconvoluted N1s spectra.

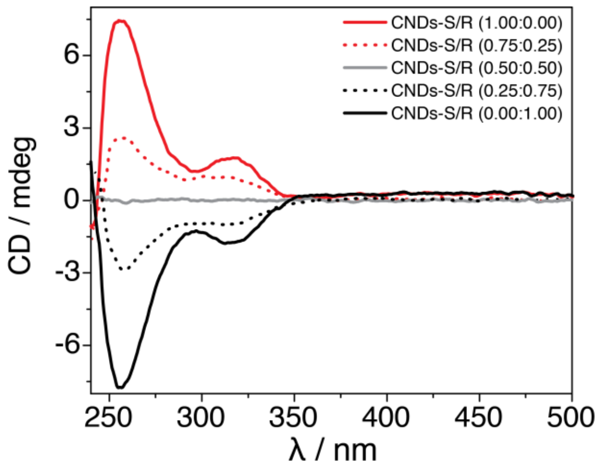

Supplementary Figure 5. **ECD spectra of CNDs-*R/S* prepared by using arginine and different molar ratios of (*S,S*)- or (*R,R*)-CHDA** – 1.00:0.00 (red line), 0.75:0.25 (red dashed line), 0.50:0.50 (gray line), 0.25:0.75 (black dashed line) and 0.00:1.00 (black line).

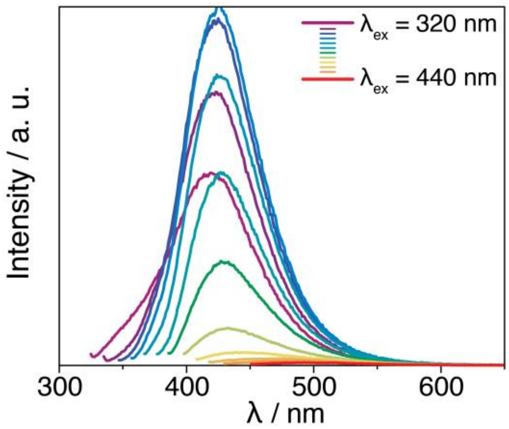

Supplementary Figure 6. **FL emission spectra of CNDs-*S***. Experiments performed in water at 298 K.

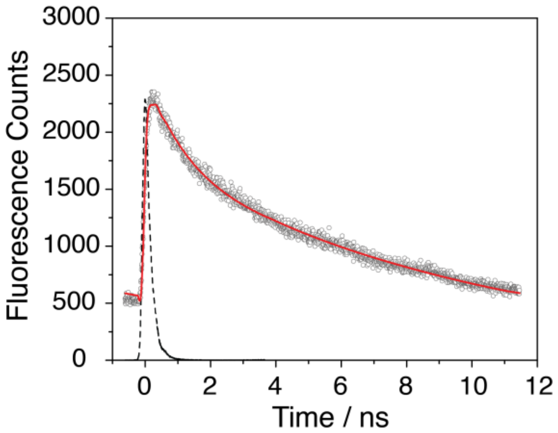

Supplementary Figure 7. **FL time decay curve**. Aqueous solution of CNDs-*S* (black dashed line is instrument response). Analogous curve was measured for the CNDs-*R* solution.

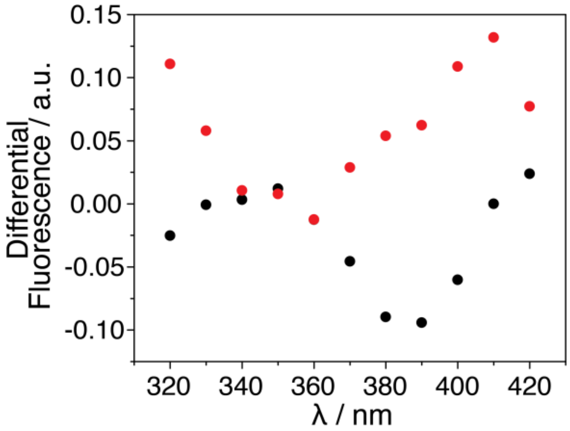

Supplementary Figure 8. **FDCD spectra of CNDs-*S* and CNDs-*R*.** FDCD data collected above 465 nm at the angle θ = -15°, symmetric with respect to that reported in the main text.

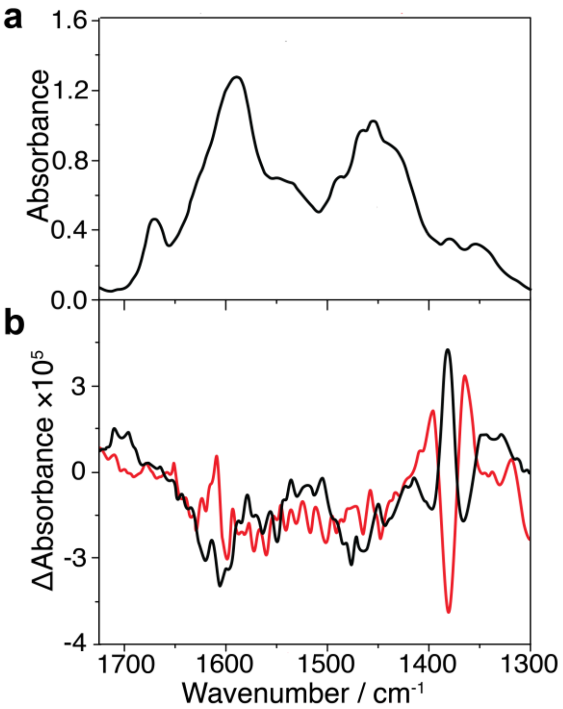

Supplementary Figure 9. **Experimental IR and VCD spectra**. Acquired (**a**) IR and (**b**) VCD spectra for CNDs-*R* (black trace) and CNDs-*S* (red trace) referenced to D_2O. The spectra were then referenced to eliminate artefacts as described in Supplementary Methods.[1]

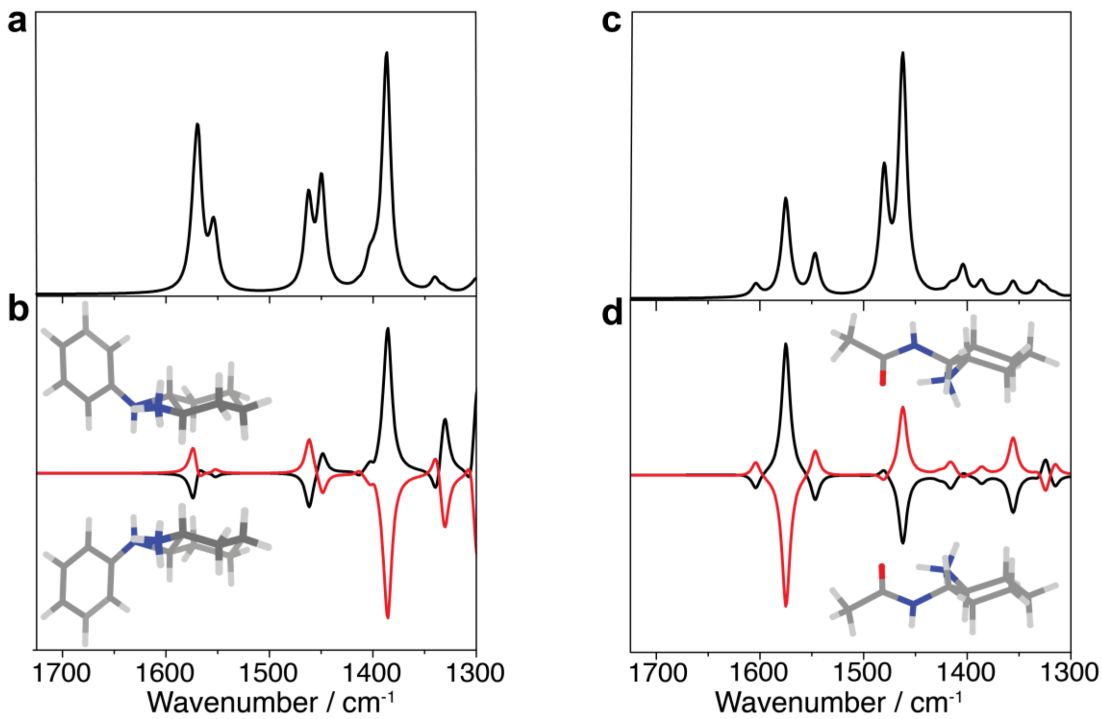

Supplementary Figure 10. **Calculated IR and VCD spectra**. DFT calculated (**a**,**c**) IR and (**b**,**d**) VCD spectra for selected cyclohexanediamine fragments, performed using the B3PW91 functional and a 6-31G** basis set, water PCM solvation.

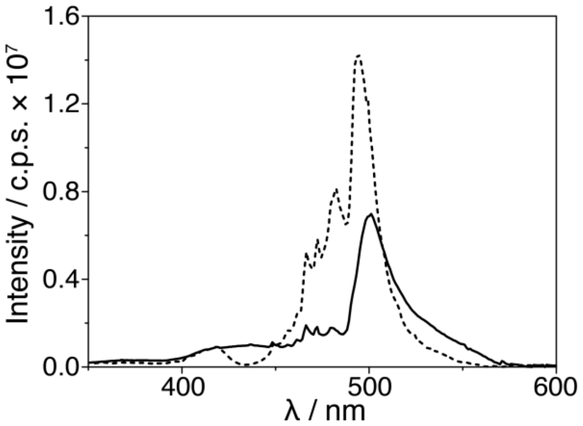

Supplementary Figure 11. **RLS spectra**. Water solution of H_4TSPP^{2-} (6.0 μM) at pH 2.5 in the presence of CNDs (black line) and in the absence of CNDs (dashed line).

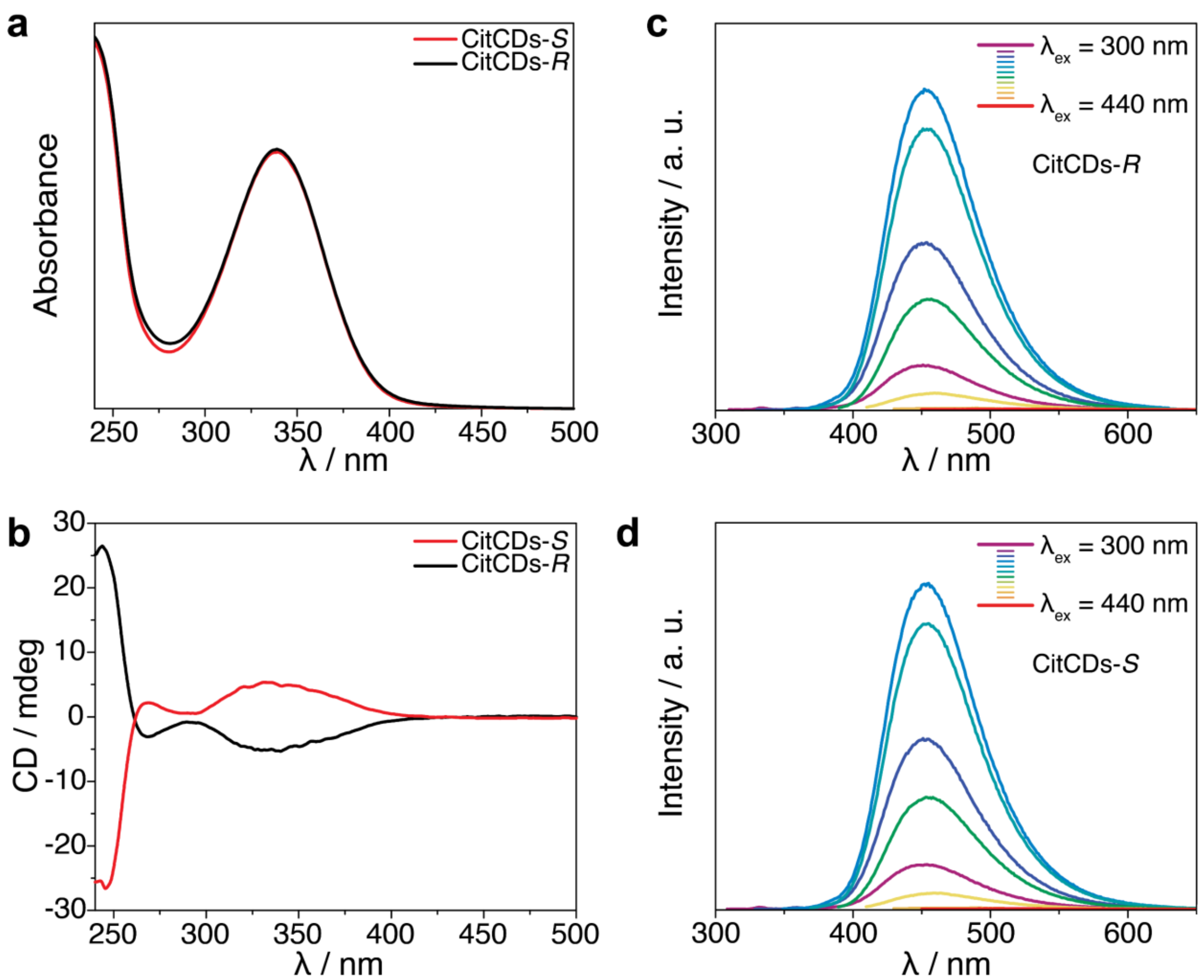

Supplementary Figure 12. **Photophysical and (chiro)optical characterization of CitCNDs.** Experiments performed in water at 298 K. (**a**) UV-Vis spectra of CitCNDs-*S* (red line) and CitCNDs-*R* (black line); (**b**) ECD spectra of CitCNDs-*S* (red line) and CitCNDs-*R* (black line); (**c,d**) FL emission spectra of CitCNDs-*R* and -*S*.

Supplementary Table

Supplementary Table 1. Fitting parameters of the fluorescence decay curves.

Sample	**A_1**	**τ_1** $\pm$ 0.1 (ns)	**A_2**	**τ_2** $\pm$ 0.1 (ns)	**τ_{ave}** (ns)
CNDs-*S*	0.27	1.2	0.73	10.8	8.2
CNDs-*R*	0.34	1.2	0.66	10.3	7.2

Supplementary Methods

Reagents and solvents were bought from Sigma-Aldrich, TCI, VWR Int. and used as received, unless otherwise specified. The two *trans*-1,2-diaminocyclohexanes were purchased from Sigma-Aldrich (346721 and 346713), TSPP was either bought as hydrate from TCI Chemicals (A5013) or prepared as tetrasodium salt following a literature procedure[2]. **Dialysis tubes** with molecular weight cut-off 1 KDa were bought from Spectrum Labs. **Ultrapure fresh water** was obtained from a water purification system (>18MΩ·cm Milli-Q, Millipore).

Microwave synthesis was performed on a CEM Discover-SP.

Fourier-Transform Infrared spectra (KBr) were recorded on a Perkin Elmer 2000 spectrometer.

Atomic Force Microscopy (AFM) images were obtained with a Nanoscope IIIa, VEECO Instruments. As a general procedure to perform AFM analyses, tapping mode with a HQ:NSC19/ALBS probe (80kHz; 0.6 N/m) (MikroMasch) from drop cast of samples in an aqueous solution (concentration of few mg/mL) on a mica substrate was performed. The obtained AFM-images were analyzed in Gwyddion 2.35. The statistical analysis was performed on about one hundred nanoparticles and the average size was calculated from the size histogram with curve fit to the data using a Gaussian model.

X-Ray Photoemission Spectroscopy (XPS) spectra were measured on a SPECS Sage HR 100 spectrometer using a non-monochromatized Mg-Kα radiation of 1253.6 eV and 250 W, in an ultra-high vacuum chamber at pressure below 8×10^{-7} mbar. For each analysis, an aqueous solution (*ca*. 3 mg/mL) of material were deposited on a gold thin film. The calibration was done using the 3d5/2 line of Ag. Survey and high-resolution spectra were collected with pass energy of 30 and 15 eV and 0.5 and 0.15 eV/step, respectively. CasaXPS 2.3.17 PR1.1 and Avantage (Thermo Fisher Scientific) software were used for data processing and fitting. Curve fittings of the C1s and N1s spectra were realized using a Gaussian-Lorentzian peak shape after performing a Shirley background correction, to finally obtain the relative percentage of each type of bond inside the analyzed sample.

UV-Vis spectra were recorded on a PerkinElmer Lambda 35 UV-Vis spectrophotometer.

Fluorescence spectra were recorded on a Varian Cary Eclipse Fluorescence Spectrophotometer. All the spectra were recorded at room temperature using 10 mm path-length cuvettes. The relative quantum yield measurements were performed using quinine sulphate in 0.10 M H_2SO_4 as reference (literature quantum yield 0.54).[3]

Time-resolved Fluorescence. A duplicated tunable femtosecond (100 fs) Ti:Sa laser (Spectra Physics MaiTai DeepSee - Radiantis Inspire Blue), set at 350 nm, was used to excite fluorescence emission. The emitted signal at 450 nm was collected, through a monochromator (Oriel Cornerstone) with focal length of 280 mm, by a multichannel-plate photomultiplier (Hamamatzu R1645U-01, 100 ps rising-time). The electronic equipment necessary for pre-amplification, constant-fraction discrimination and laser pulse synchronization in the time-correlated-single-photon counting (TCSPC) method was realized with EG&G devices. By using nonlinear least-squares iterative reconvolution procedures a time resolution better than 50 ps was obtained.
Fluorescence time decay curve was fitted according to the following multiexponential relation, Supplementary Equation 1:

$$I(t) = I_0 \sum_i A_i exp(-t/\tau_i)$$

in which $I(t)$ is the fluorescence decay curve, I_0 is the intensity at time zero and A_i is the amplitude of the ith component ($\sum_i A_i = 1$) with lifetime τ_i.

Differential Fluorescence. Fluorescence detected circular dichroism measurements were carried out by exploiting the circularly polarized light exiting from a JASCO J500A spectropolarimeter (with resolution of 8 nm) to illuminate the sample, whereas the detection apparatus was modified as sketched in Supplementary Figure 13. In particular, the circularly polarized light was focused onto the sample, contained in a 4-windows quartz cuvette with path 1 cm, by a quartz lens (L1, focal length 150 mm). The illuminating cylindrical spot was 2 mm wide. A glass lens (L2, focal length 50 mm) was placed after the sample to obtain the image of the illuminated sample volume at the photomultiplier (PM, Hamamatzu R376). In order to reject the excitation light from the collected signal, a longpass filter (LP) with cut-off at 465 nm (optical density higher than 3 below 425 nm and transmission 90% above 500 nm) was placed just before the photomultiplier and all the detection optics was placed on a guide mounted on a goniometer to collect the emission off the transmitted beam. The angle, θ, between the collected signal and the transmitted beam was set at 15° and at -15°, in order to check out for eventual artefacts due to fluorescence polarization effects. At this angle, no portion of the transmitted beam passed through the collecting lens L2.

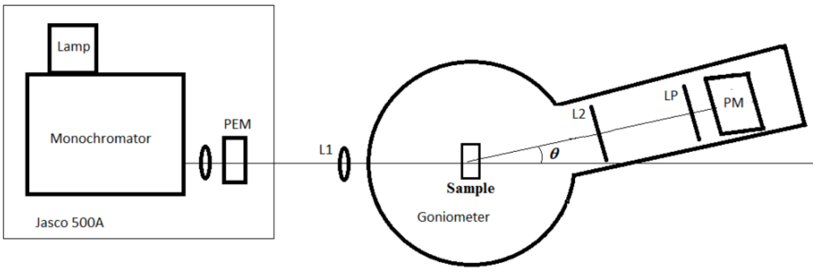

Supplementary Figure 13. **FDCD experimental set-up**. Sketch of the home-made experimental set-up for the fluorescence detected circular dichroism measurements.

The high tension of the photomultiplier was controlled in between 650 and 850 V to maintain the current constant in all the wavelength range investigated and its pre-amplified signal was analyzed by a lock-in amplifier (Stanford SR510). Due to the low signal-to-noise ratio, the lock-in integration time constant was set at 30 s and the spectra reported are the average of five repeated measurements.
The concentration of the CNDs solutions was 0.16 mg/mL for both the *R*- and *S*-moieties and the pH of the solution was 9. The baseline for the fluorescence detected circular dichroism signal was obtained by using a sodium fluorescein aqueous solution at the concentration of 2×10^{-5} M.

Electronic Circular Dichroism. CD spectra were recorded using Jasco J-810 at room temperature. A quartz cuvette with a 1 cm path length was used for all CD experiments. Conditions were as follows: scanning rate 50 nm/min, data pitch 0.2 nm, D.I.T 2 s. Each CD spectrum was an average of at least five scans.

Resonance Light Scattering. RLS measurements were acquired using a spectrophotometer FL11 Jobin Yvon Horiba, in synchronous mode. A four-face quartz cuvette with a 1 cm path length was used.

Vibrational Circular Dichroism. Vibrational circular dichroism (VCD) spectra were recorded on a Bruker PMA 50 accessory coupled to a Tensor 27 Fourier transform infrared spectrometer. A photoelastic modulator (Hinds PEM 90) set at 1/4 retardation was used to modulate the handedness of the circular polarized light. Demodulation was performed by a lock-in amplifier (SR830 DSP). An optical low-pass filter (< 1800 cm^{-1}) in front of the photoelastic modulator was used to enhance the signal/noise ratio. The sample was dissolved in D_2O (*ca*. 5 mg in 50 mL). The solution was measured as a film of *ca*. 13 µm thickness sandwiched between two CaF_2 windows. The average of the measured VCD spectra of the two enantiomers served as the reference and was subtracted from the VCD spectra of pure enantiomers in order to eliminate artefacts.[1] Note that this procedure automatically produces mirror image spectra. For both enantiomers 24000 scans at 4 cm^{-1} resolution were averaged.

DFT calculations. The simulation of VCD spectra started by constructing probable cyclohexanediamine fragments, with information extracted from experimental data. Preliminary conformational searches of cyclohexanediamine fragments (neutral or protonated) were performed using semi-empirical level (PM3)[4] and the conformational geometries were then optimized using DFT (B3LYP/6-31G*)[5–7], using the Spartan program package.[8] The most stable conformers were then used as starting geometries for optimization and frequency (=vcd) calculations (B3LYP/6-31G**), in water (PCM solvation)[9], using Gaussian software.[10] Finally, the selected fragments conformers were used for VCD calculations using the B3PW91[7,11] hybrid functional and 6-31G** basis set[12], in water. Vibrational frequencies were scaled by a factor of 0.95. IR and VCD spectra were constructed from calculated dipole and rotational strengths

assuming Lorentzian band shape with a half-width at half-height = 5 cm^{-1}. Description of calculated vibrations was made from animations of the modes and spectra with GaussView 5.08.[13]

Synthesis of CNDs-*S* and -*R*. Chiral CNDs were obtained via microwave irradiation of an aqueous solution of L-Arginine (Arg) and (*R,R*)-(—)-cyclohexanediamine or (*S,S*)-(+)-cyclohexanediamine (1:1 mol/mol). Typically, Arg (87.0 mg), (*R,R*)-(—)-cyclohexanediamine or (*S,S*)-(+)-cyclohexanediamine (57.0 mg) and Milli-Q water (100.0 μL) were heated at 240 °C, 70-110 psi and 200 W for 180 seconds. In the process of microwave heating, the color changed from yellow to dark brown as a result of formation of CNDs. The reaction mixture was then diluted with a few milliliters of water and the solution was filtered through a 0.1 μm microporous membrane separating a deep yellow solution that was dialyzed against pure water through a dialysis membrane for 2 days. The aqueous solution was then lyophilized giving a brownish solid (CNDs-*R*: 20.5 mg; CNDs-*S*: 22.0 mg).

CitCNDs-*S* and CitCNDs-*R* were obtained via microwave irradiation of an aqueous solution of citric acid (CA) and (*R,R*)-(—)-cyclohexanediamine or (*S,S*)-(+)-cyclohexanediamine following a modified literature procedure[14]. Briefly, CA (75.0 mg), (*R,R*)-(—)-cyclohexanediamine or (*S,S*)-(+)-cyclohexanediamine (75.0 mg) and Milli-Q water (250.0 μL) were heated for 5 minutes and the temperature was controlled to maintain a pressure of 210 psi. In the process of microwave heating, the color changed from yellow to brown as a result of the formation of CNDs. The reaction mixture was then diluted with a few milliliters of water and the solution was filtered through a 0.1 μm microporous membrane separating a deep yellow solution, which was then dialyzed against pure water through a dialysis membrane for 2 days.

Chiral Supramolecular Assemblies. CND stock solution was prepared by dissolving 1.9 mg in 2.5 mL of ultrapure water obtained from Elga Purelab Flex system by Veolia; the pH of the solution was 9. The porphyrin selected for our study is the tetranionic *meso*-tetrakis(4-sulfonatophenyl)porphyrin (H_2TSPP^{4-}). The stock solution of porphyrin was prepared by dissolving small amount of solid in ultrapure water at pH 7 in order to obtain concentration about 2×10^{-4} M, checked by spectrophotometer Jasco V-630 using $\varepsilon = 4.8 \times 10^{-5}$ M^{-1} cm^{-1} at $\lambda = 413$ nm.
The sample solutions were achieved mixing CND solution and H_2TSPP^{4-} at pH = 6.5. After 30 minutes incubation, the pH was decreased to 2.5 in order to induce the formation of J-aggregates.

Supplementary References

1. Nafie, L. A. *Vibrational Optical Activity: Principles and Applications*. (John Wiley & Sons, 2011).
2. Dong, Z. & Scammells, P. J. New methodology for the N-demethylation of opiate alkaloids. *J. Org. Chem.* **72,** 9881–9885 (2007).
3. Würth, C., Grabolle, M., Pauli, J., Spieles, M. & Resch-Genger, U. Relative and absolute determination of fluorescence quantum yields of transparent samples. *Nat. Protoc.* **8,** 1535–50 (2013).
4. Stewart, J. J. P. Optimization of parameters for semiempirical methods II. Applications. *J. Comput. Chem.* **10,** 221–264 (1989).
5. Becke, A. D. Density-functional exchange-energy approximation with correct asymptotic behavior. *Phys. Rev. A* **38,** 3098–3100 (1988).
6. Lee, C., Yang, W. & Parr, R. G. Development of the Colle-Salvetti correlation-energy formula into a functional of the electron density. *Phys. Rev. B* **37,** 785–789 (1988).
7. Becke, A. D. Density-functional thermochemistry. III. The role of exact exchange. *J. Chem. Phys.* **98,** 5648–5652 (1993).
8. Spartan ’14; Wavefuntion, Inc.; Irvine, CA.
9. Tomasi, J., Mennucci, B. & Cammi, R. Quantum Mechanical Continuum Solvation Models. *Chem. Rev.* **105,** 2999–3094 (2005).
10. Frisch, M. J. *et al.* Gaussian 09, Revision D 0.1. (2009).
11. Perdew, J. P. *et al.* Atoms, molecules, solids, and surfaces: Applications of the generalized gradient approximation for exchange and correlation. *Phys. Rev. B* **46,** 6671–6687 (1992).
12. Ditchfield, R., Hehre, W. J. & Pople, J. A. Self-Consistent Molecular-Orbital Methods. IX. An Extended Gaussian-Type Basis for Molecular-Orbital Studies of Organic Molecules. *J. Chem. Phys.* **54,** 724–728 (1971).
13. Dennington, Roy; Keith, Todd A.; Millam, J. M. GaussView. (2009).
14. Strauss, V. *et al.* Carbon Nanodots: Toward a Comprehensive Understanding of Their Photoluminescence. *J. Am. Chem. Soc.* **136,** 17308–17316 (2014).
